# 9,10-Dihydro-7*H*-benzo[*de*]imidazo[2,1-*a*]isoquinolin-7-one

**DOI:** 10.1107/S1600536812022921

**Published:** 2012-05-26

**Authors:** Yu Mei Chen, Jian Chao Shi

**Affiliations:** a Department of Physics and Chemistry, Henan Polytechnic University, Jiaozuo, Henan 454000, People’s Republic of China

## Abstract

In the title compound, C_14_H_10_N_2_O, all non-H atoms are essentially coplanar (r.m.s. deviation = 0.013 Å). The crystal structure is stabilized by π–π stacking inter­actions [centroid–centroid distance = 3.506 (3) Å].

## Related literature

For the use of rigid ligands in the formation of metal-organic coordination polymers, see: Chen *et al.* (2006 ▶); Yang *et al.* (2009 ▶).
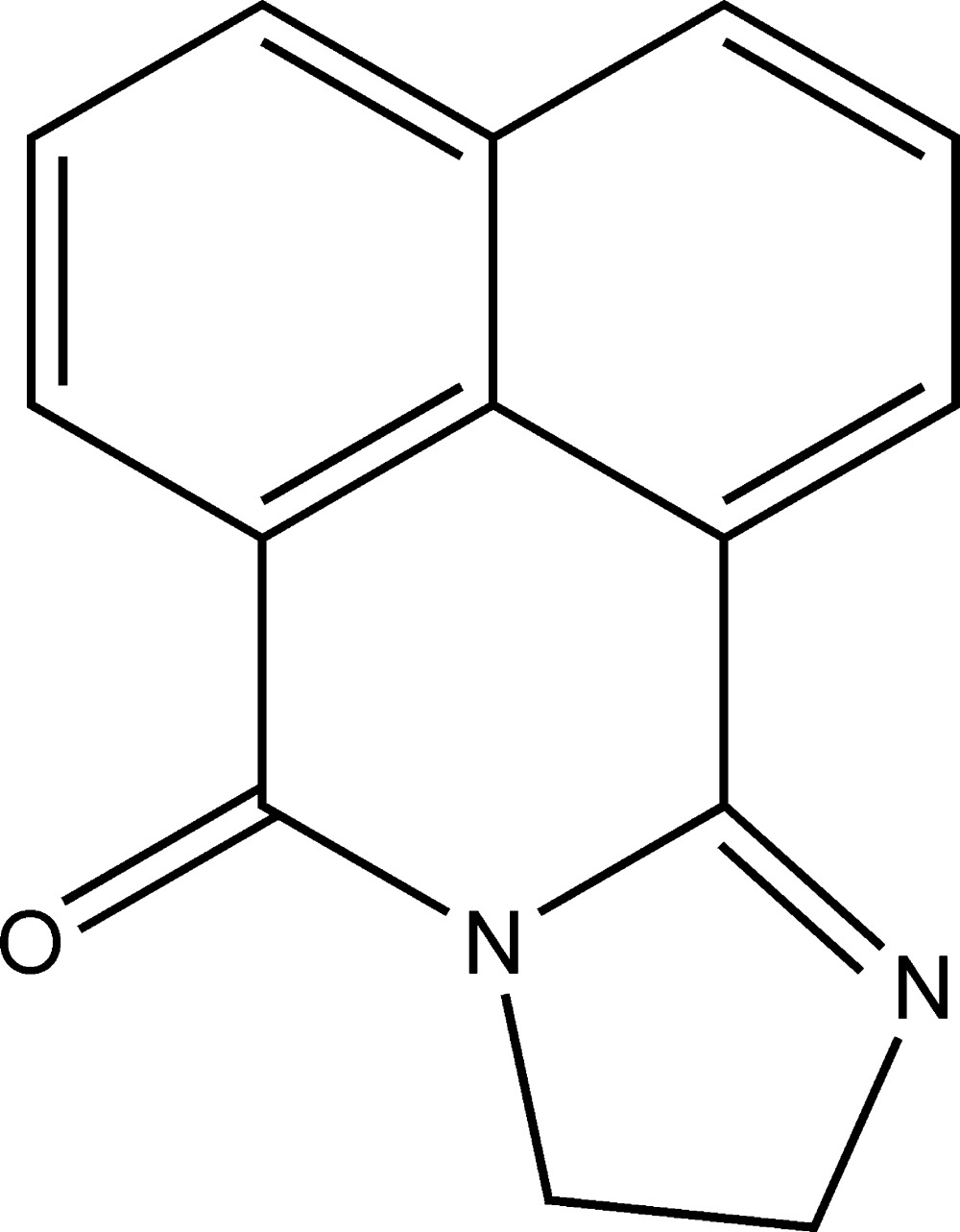


## Experimental

### 

#### Crystal data

C_14_H_10_N_2_O
*M* *_r_* = 222.24
Orthorhombic, 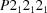

*a* = 4.4949 (2) Å
*b* = 14.9891 (9) Å
*c* = 15.1357 (8) Å
*V* = 1019.76 (9) Å^3^
*Z* = 4
Mo *K*α radiation
μ = 0.09 mm^−1^
*T* = 296 K
0.20 × 0.05 × 0.05 mm

#### Data collection

Bruker SMART APEXII CCD area-detector diffractometer
Absorption correction: multi-scan (*SADABS*; Sheldrick, 1996 ▶) *T*_min_ = 0.982, *T*_max_ = 0.995
8736 measured reflections
1837 independent reflections
1207 reflections with *I* > 2σ(*I*)
*R*_int_ = 0.046

#### Refinement

*R*[*F* ^2^ > 2σ(*F* ^2^)] = 0.075
*wR*(*F* ^2^) = 0.202
*S* = 1.01
1837 reflections
154 parameters
1 restraint
H-atom parameters constrained
Δρ_max_ = 0.27 e Å^−3^
Δρ_min_ = −0.33 e Å^−3^

### 

Data collection: *APEX2* (Bruker, 2008 ▶); cell refinement: *SAINT* (Bruker, 2008 ▶); data reduction: *SAINT*; program(s) used to solve structure: *SHELXS97* (Sheldrick, 2008 ▶); program(s) used to refine structure: *SHELXL97* (Sheldrick, 2008 ▶); molecular graphics: *SHELXTL* (Sheldrick, 2008 ▶) and *PLATON* (Spek, 2009 ▶); software used to prepare material for publication: *SHELXTL*.

## Supplementary Material

Crystal structure: contains datablock(s) I, global. DOI: 10.1107/S1600536812022921/bx2409sup1.cif

Structure factors: contains datablock(s) I. DOI: 10.1107/S1600536812022921/bx2409Isup2.hkl

Additional supplementary materials:  crystallographic information; 3D view; checkCIF report

## Figures and Tables

**Fig. 1.**
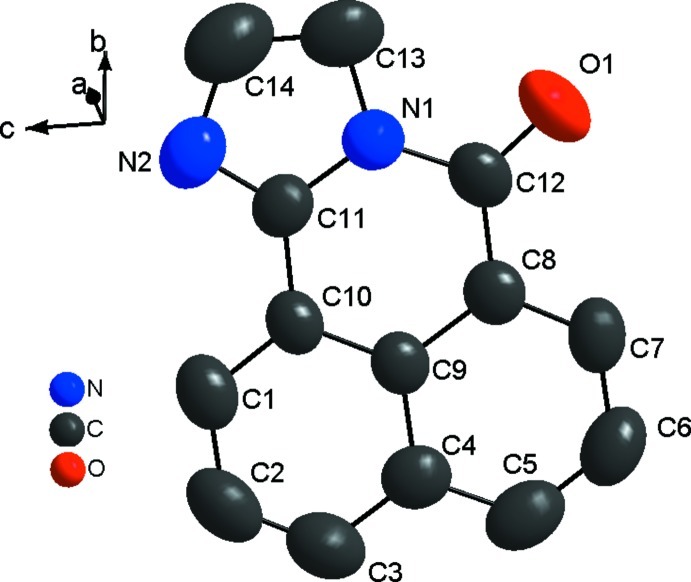
The molecular structure of the title compound. Displacement ellipsoids are drawn at the 30% probability level.

## supplementary crystallographic information

### Comment

The title compound, C_14_H_10_N_2_O, (I) can be used as a rigid ligand to form metal-organic coordination polymers, such as [Ag(C_14_H_10_N_2_O)(NO_3_)]_n_, [Ag(C_14_H_10_N_2_O)_2_(NO_3_)]_n_, [Ag(C_14_H_10_N_2_O)_2_ (BF_4_)]_n _(Yang *et al.*, 2009) and [Cu_2_(CH_3_COO)_4_(C_14_H_10_N_2_O)_2_]_n_ (Chen *et al.*, 2006). However, the crystal structure of 9,10-dihydro-7*H*-benzo[de]imidazo[2,1-*a*]-isoquinolin-7-one have not been reported so far. We report herein the synthesize and the crystal structure of (I). In the title molecule , C_14_H_10_N_2_O, all non-H atoms are essentially coplanar (r.m.s. 0.013 Å). The crystal structure is stabilized by π–π stacking interactions (centroid -centroid distance 3.506 (3)Å, Cg =C4/C5/C6/C7/C8/C9 ; Cg^i^ =C4/C5/C6/C7/C8/C9 ; symmetry code (i) x-1, y, z)

### Experimental

White prism-shaped single crystals of 9,10-dihydro-7*H*-benzo[de]imidazo[2,1-*a*]-isoquinolin-7-one were initially obtained from the hydrothermal reaction of Naphthalene-1,8-dicarboxylic anhydride (0.3 g), ethylenediamine (5 ml) and H_2_O (10 ml) using Teflon lined bomb at 160°C for 5 days and then cooled to room temperature. A few single crystals suitable for X-ray diffraction analysis were obtained.

### Refinement

Constraint instruction 'DELU 0.01 C14 N2' was used in the refinement. The final difference map shows that the highest peak is 0.27 e/Å^3^ at 1.55 Å from O(1), while the deepest hole is -0.33 e/Å^3^ at 0.16 Å from H(13B). H atoms were placed in geometrically calculated positions with C—H distances in the range 0.93-0.97Å and were refined using a riding model, with U_iso_(H)=1.2U_eq_(C). Friedel pairs (715) were merged.

### Crystal data

**Table:**

C_14_H_10_N_2_O	*F*(000) = 464
*M**_r_* = 222.24	*D*_x_ = 1.448 Mg m^−^^3^
Orthorhombic, *P*2_1_2_1_2_1_	Mo *K*α radiation, λ = 0.71073 Å
Hall symbol: P 2ac 2ab	Cell parameters from 946 reflections
*a* = 4.4949 (2) Å	θ = 2.7–19.8°
*b* = 14.9891 (9) Å	µ = 0.09 mm^−^^1^
*c* = 15.1357 (8) Å	*T* = 296 K
*V* = 1019.76 (9) Å^3^	Prism, colourless
*Z* = 4	0.20 × 0.05 × 0.05 mm

### Data collection

**Table:**

Bruker SMART APEXII CCD area-detector diffractometer	1837 independent reflections
Radiation source: fine-focus sealed tube	1207 reflections with *I* > 2σ(*I*)
Graphite monochromator	*R*_int_ = 0.046
Detector resolution: 83.33 pixels mm^-1^	θ_max_ = 25.5°, θ_min_ = 1.9°
ω scans	*h* = −5→5
Absorption correction: multi-scan (*SADABS*; Sheldrick, 1996)	*k* = −18→16
*T*_min_ = 0.982, *T*_max_ = 0.995	*l* = −18→16
8736 measured reflections	

### Refinement

**Table:**

Refinement on *F*^2^	Primary atom site location: structure-invariant direct methods
Least-squares matrix: full	Secondary atom site location: difference Fourier map
*R*[*F*^2^ > 2σ(*F*^2^)] = 0.075	Hydrogen site location: inferred from neighbouring sites
*wR*(*F*^2^) = 0.202	H-atom parameters constrained
*S* = 1.01	*w* = 1/[σ^2^(*F*_o_^2^) + (0.0818*P*)^2^ + 0.9931*P*] where *P* = (*F*_o_^2^ + 2*F*_c_^2^)/3
1837 reflections	(Δ/σ)_max_ < 0.001
154 parameters	Δρ_max_ = 0.27 e Å^−^^3^
1 restraint	Δρ_min_ = −0.33 e Å^−^^3^
1 constraint	

### Special details

**Table:**

Geometry. All e.s.d.'s (except the e.s.d. in the dihedral angle between two l.s. planes) are estimated using the full covariance matrix. The cell e.s.d.'s are taken into account individually in the estimation of e.s.d.'s in distances, angles and torsion angles; correlations between e.s.d.'s in cell parameters are only used when they are defined by crystal symmetry. An approximate (isotropic) treatment of cell e.s.d.'s is used for estimating e.s.d.'s involving l.s. planes.
Refinement. Refinement of *F*^2^ against ALL reflections. The weighted *R*-factor *wR* and goodness of fit *S* are based on *F*^2^, conventional *R*-factors *R* are based on *F*, with *F* set to zero for negative *F*^2^. The threshold expression of *F*^2^ > σ(*F*^2^) is used only for calculating *R*-factors(gt) *etc*. and is not relevant to the choice of reflections for refinement. *R*-factors based on *F*^2^ are statistically about twice as large as those based on *F*, and *R*- factors based on ALL data will be even larger.

### Fractional atomic coordinates and isotropic or equivalent isotropic displacement parameters (Å^2^)

**Table:**

	*x*	*y*	*z*	*U*_iso_*/*U*_eq_	
N1	0.8099 (9)	0.5958 (3)	0.5858 (3)	0.0586 (11)	
C9	0.4344 (11)	0.4484 (3)	0.5830 (3)	0.0526 (11)	
C10	0.5848 (10)	0.4693 (3)	0.6615 (3)	0.0537 (11)	
C12	0.6647 (12)	0.5790 (3)	0.5059 (3)	0.0614 (14)	
C11	0.7765 (11)	0.5470 (3)	0.6614 (3)	0.0556 (11)	
C5	0.0907 (13)	0.3543 (4)	0.4989 (4)	0.0772 (17)	
H5	−0.0366	0.3055	0.4963	0.093*	
C1	0.5498 (12)	0.4169 (4)	0.7347 (3)	0.0692 (14)	
H1	0.6487	0.4308	0.7868	0.083*	
O1	0.7130 (11)	0.6295 (2)	0.4429 (2)	0.0886 (14)	
C8	0.4680 (11)	0.5014 (3)	0.5046 (3)	0.0557 (12)	
C4	0.2423 (12)	0.3730 (3)	0.5796 (3)	0.0620 (13)	
C7	0.3183 (12)	0.4788 (4)	0.4287 (3)	0.0673 (14)	
H7	0.3457	0.5130	0.3781	0.081*	
N2	0.9310 (11)	0.5775 (3)	0.7269 (3)	0.0773 (13)	
C3	0.2118 (14)	0.3213 (4)	0.6554 (4)	0.0774 (16)	
H3	0.0863	0.2720	0.6547	0.093*	
C13	1.0057 (13)	0.6648 (3)	0.6011 (4)	0.0778 (17)	
H13A	0.9115	0.7223	0.5921	0.093*	
H13B	1.1794	0.6604	0.5633	0.093*	
C6	0.1267 (13)	0.4058 (4)	0.4255 (4)	0.0805 (17)	
H6	0.0242	0.3924	0.3738	0.097*	
C2	0.3623 (14)	0.3418 (4)	0.7303 (4)	0.0825 (18)	
H2	0.3413	0.3055	0.7797	0.099*	
C14	1.0800 (16)	0.6516 (4)	0.6897 (5)	0.100 (2)	
H14A	1.0297	0.7048	0.7230	0.120*	
H14B	1.2931	0.6425	0.6945	0.120*	

### Atomic displacement parameters (Å^2^)

**Table:**

	*U*^11^	*U*^22^	*U*^33^	*U*^12^	*U*^13^	*U*^23^
N1	0.061 (3)	0.056 (2)	0.060 (3)	0.003 (2)	0.006 (2)	−0.006 (2)
C9	0.052 (3)	0.054 (3)	0.051 (3)	0.013 (2)	0.011 (2)	−0.003 (2)
C10	0.050 (2)	0.061 (3)	0.051 (3)	0.012 (2)	0.006 (2)	0.002 (2)
C12	0.068 (3)	0.054 (3)	0.062 (3)	0.016 (3)	0.011 (3)	0.002 (2)
C11	0.047 (3)	0.061 (3)	0.059 (3)	0.010 (3)	0.005 (2)	−0.008 (2)
C5	0.057 (3)	0.079 (4)	0.095 (5)	−0.001 (3)	0.011 (3)	−0.028 (3)
C1	0.066 (3)	0.083 (4)	0.059 (3)	0.018 (3)	0.007 (3)	0.005 (3)
O1	0.118 (3)	0.074 (2)	0.074 (2)	0.006 (3)	0.021 (3)	0.021 (2)
C8	0.050 (3)	0.058 (2)	0.060 (3)	0.014 (2)	0.003 (2)	0.001 (2)
C4	0.051 (3)	0.060 (3)	0.075 (3)	0.009 (3)	0.011 (3)	−0.008 (3)
C7	0.067 (3)	0.081 (4)	0.054 (3)	0.015 (3)	0.000 (3)	−0.004 (3)
N2	0.077 (3)	0.081 (3)	0.074 (3)	0.003 (3)	−0.007 (3)	−0.014 (2)
C3	0.070 (4)	0.067 (3)	0.095 (4)	0.006 (3)	0.023 (3)	0.007 (3)
C13	0.068 (4)	0.059 (3)	0.106 (5)	0.011 (3)	0.019 (3)	−0.020 (3)
C6	0.072 (4)	0.098 (4)	0.072 (4)	0.005 (4)	−0.008 (3)	−0.023 (4)
C2	0.081 (4)	0.084 (4)	0.083 (4)	0.014 (3)	0.019 (3)	0.024 (3)
C14	0.083 (4)	0.097 (5)	0.120 (6)	0.010 (4)	0.015 (4)	−0.041 (4)

### Geometric parameters (Å, º)

**Table:**

N1—C11	1.367 (5)	C1—H1	0.9300
N1—C13	1.377 (6)	C8—C7	1.375 (7)
N1—C12	1.397 (6)	C4—C3	1.391 (7)
C9—C10	1.402 (6)	C7—C6	1.392 (7)
C9—C4	1.423 (6)	C7—H7	0.9300
C9—C8	1.436 (6)	N2—C14	1.414 (8)
C10—C1	1.367 (7)	C3—C2	1.355 (8)
C10—C11	1.448 (6)	C3—H3	0.9300
C12—O1	1.236 (5)	C13—C14	1.396 (8)
C12—C8	1.462 (7)	C13—H13A	0.9700
C11—N2	1.295 (6)	C13—H13B	0.9700
C5—C6	1.363 (8)	C6—H6	0.9300
C5—C4	1.426 (7)	C2—H2	0.9300
C5—H5	0.9300	C14—H14A	0.9700
C1—C2	1.408 (8)	C14—H14B	0.9700
			
C11—N1—C13	109.4 (4)	C9—C4—C5	118.5 (5)
C11—N1—C12	125.2 (4)	C8—C7—C6	121.7 (5)
C13—N1—C12	125.4 (5)	C8—C7—H7	119.2
C10—C9—C4	120.1 (4)	C6—C7—H7	119.2
C10—C9—C8	121.7 (4)	C11—N2—C14	103.1 (5)
C4—C9—C8	118.2 (4)	C2—C3—C4	121.0 (6)
C1—C10—C9	120.2 (5)	C2—C3—H3	119.5
C1—C10—C11	122.1 (5)	C4—C3—H3	119.5
C9—C10—C11	117.8 (4)	N1—C13—C14	102.0 (5)
O1—C12—N1	118.4 (5)	N1—C13—H13A	111.4
O1—C12—C8	125.7 (5)	C14—C13—H13A	111.4
N1—C12—C8	116.0 (4)	N1—C13—H13B	111.4
N2—C11—N1	113.1 (4)	C14—C13—H13B	111.4
N2—C11—C10	127.0 (5)	H13A—C13—H13B	109.2
N1—C11—C10	119.8 (4)	C5—C6—C7	119.3 (5)
C6—C5—C4	122.0 (6)	C5—C6—H6	120.3
C6—C5—H5	119.0	C7—C6—H6	120.3
C4—C5—H5	119.0	C3—C2—C1	121.3 (5)
C10—C1—C2	119.3 (5)	C3—C2—H2	119.3
C10—C1—H1	120.3	C1—C2—H2	119.3
C2—C1—H1	120.3	C13—C14—N2	112.4 (6)
C7—C8—C9	120.2 (5)	C13—C14—H14A	109.1
C7—C8—C12	120.3 (5)	N2—C14—H14A	109.1
C9—C8—C12	119.6 (4)	C13—C14—H14B	109.1
C3—C4—C9	118.1 (5)	N2—C14—H14B	109.1
C3—C4—C5	123.4 (6)	H14A—C14—H14B	107.9
